# Diagnostic identification of chronic insomnia using ALFF and FC features of resting-state functional MRI and logistic regression approach

**DOI:** 10.1038/s41598-022-24837-8

**Published:** 2023-01-09

**Authors:** Ning Yang, Shuyi Yuan, Chunlong Li, Wenqing Xiao, Shuangcong Xie, Liming Li, Guihua Jiang, Xiaofen Ma

**Affiliations:** 1 Department of Medical Imaging, Guangdong Second Provincial General Hospital, Guangzhou, 510317 People’s Republic of China; 2 Equipment Department, Guangdong Second Provincial General Hospital, Guangzhou, People’s Republic of China; 3 Department of Medical Imaging, The First Affiliated Hospital of Guangzhou Medical University, Guangzhou, People’s Republic of China

**Keywords:** Diagnosis, Brain imaging, Biomedical engineering, Sleep disorders

## Abstract

This study investigated whether the amplitude of low-frequency fluctuation (ALFF) and functional connectivity (FC) features could be used as potentially neurological markers to identify chronic insomnia (CI) using resting-state functional MRI and machine learning method logistic regression (LR). This study included 49 CI patients and 47 healthy controls (HC). Voxel-wise features, including the amplitude of low-frequency fluctuations (ALFF) and functional connectivity (FC), were extracted from resting-state functional magnetic resonance brain images. Then, we divided the data into two independent cohorts for training (44 CI patients and 42 HC patients), and independent validation (5 CI patients and 5 HC patients) by using logistic regression. The model was evaluated using 20 rounds of fivefold cross‑validation for training. In particular, a two-sample t-test (GRF corrected, p-voxel < 0.001, p-cluster < 0.05) was used for feature selection during the model training. Finally, single‑shot testing of the final model was performed on the independent validation cohort. A correlation analysis (Bonferroni correction, p < 0.05/4) was also conducted to determine whether the features contributing to the prediction were correlated with clinical characteristics, including the Insomnia Severity Index (ISI), Pittsburgh sleep quality index (PSQI), self-rating anxiety scale (SAS), and self-rating depression scale (SDS). Results showed that resting-state features had a discrimination accuracy of 86.40%, with a sensitivity of 93.00% and specificity of 79.80%. The area under the curve (AUC) was 0.89 (all ${P}_{\mathrm{permutation}}$< 0.001). The ALFF and FC features showed significant differences between the CI patients and HC. The regions contributing to the prediction mainly included the anterior cingulate, prefrontal cortex, orbital part of the frontal lobe, angular gyrus, cingulate gyrus, praecuneus, parietal lobe, temporal gyrus, superior temporal gyrus, and middle temporal gyrus. Furthermore, some specific functional connectivity among related regions was positively correlated with the ISI, and also negatively related to the SDS in correlation analysis. Our current study suggested that ALFF and FC in the regions contributing to diagnostic identification might serve as potential neuromarkers for CI.

## Introduction

Chronic insomnia (CI) is a common clinical disease that is characterised by difficulty in falling asleep, difficulty maintaining sleep, or early awakening lasting for at least 1 month, accompanied by daytime cognitive impairment^1,2^. CI leads to daytime fatigue, emotional disruptions, and cognitive impairment, which can result in various psychological and cognitive disorders such as depressive and anxiety disorders^3,4^. However, despite its adverse social-economic effects, the neurological causes and consequences of CI are not fully understood.

Recent advances in neuroimaging techniques have provided a powerful tool for studying the neurobiological mechanisms of CI. Resting-state functional magnetic resonance imaging (rs-fMRI) has become a powerful technique for imaging brain activity in vivo, providing a new approach for studying the mechanism of CI. Li et al.^5^ demonstrated that CI patients had lower amplitude of low-frequency fluctuation (ALFF) values in the left orbitofrontal cortex/inferior frontal gyrus, right middle frontal gyrus, left inferior parietal lobule, and bilateral cerebellum posterior lobes, with higher ALFF values in the right middle/inferior temporal lobe extended to the right occipital lobe. Dai et al.^6^ also used the ALFF method to find that CI patients had higher ALFF values in the temporal and occipital lobes, with lower ALFF values in the bilateral cerebellum. The functional connectivity (FC) is another powerful tool for studying the neurobiological mechanisms of CI. A series of studies^5,7,8^ found functional abnormalities in patients with insomnia, associated with a wide range of cortical and subcortical regions, including the reticular ascending activation systems, islands, amygdala, cingulate cortex, hippocampus, frontal cortex, and caudate nucleus. From the perspective of a functional connection network^9,10^, these areas primarily consist of the default mode network (DMN), salience network (SN), affective network (AN), central executive network (CEN), and subcortical area (SUB).

While the FC and ALFF features are valuable in insomnia research, the relevant studies have often reported level differences between patients with CI and healthy controls, and doctors need to make judgements at the individual level for diagnosis and treatment. Therefore, in order for neuroimaging studies to better serve clinical diagnosis, individual-level diagnosis and prediction are required. In recent years, machine learning methods have been widely used in neuroimaging data analysis, and can extract effective information from neuroimaging data, find neurological markers based on brain image data, and distinguish patients with neuropsychiatric diseases from normal people at the individual level. A related study^11^ on the classification and prediction of patients with mood disorders based on fMRI was published. The results showed that their applied classification algorithm (support vector machine, SVM) could better diagnose patients with mood disorders and accurately predict the drug response of complex patients. Mao et al.^12^ used a logistic regression method and combined multiple neuroimaging data for the diagnosis of Alzheimer’s disease and mild cognitive impairment. Their results suggested that the use of multiple neuroimaging markers can improve the diseases diagnosis performance.

To date, it is still unclear whether the FC and ALFF features could be used as neurological markers for the diagnosis of CI patients at the individual level, and few studies have applied machine learning methods to the diagnosis of CI. Deep learning was applied on a set of 57 EEG features to accurately distinguish between patients with insomnia and healthy controls^13^. The classifier had an accuracy of up to 86%. Li et al.^14^ suggested that the functional connectivity strength (FCS) could be used as potential neuromarkers for the classification of CI patients and healthy controls (HC) using the SVM method. The classification accuracy was 81.5%. Ramiro et al.^15^ used a logistic regression (LR) method trained with a set of similarity measures to distinguish between control and insomnia subjects. The LR model classified controls and insomnia subjects with an accuracy of 81%.

In the present study, voxel-wise features such as the ALFF and FC were extracted from resting-state functional magnetic resonance (MR) brain images. The machine learning method LR^16,17^ was used to classify the CI patients and HC to investigate whether these features could be used as potentially neurological markers for the classification of CI. In particular, a two-sample t-test (GRF corrected, p-voxel < 0.001, p-cluster < 0.05) was used to perform feature selection during model training.

## Materials and methods

### Participants

This prospective study was approved by the ethics committee of the Guangdong Second Provincial General Hospital and all the participants provided written informed consent after they were provided with a complete description of the study. We confirmed that all methods were carried out in accordance with relevant guidelines and regulations. Forty-nine patients with CI (21 males and 28 females, with a mean age ± standard deviation of 39.27 ± 11.00) were recruited from the Guangdong Second Provincial General Hospital.

The following inclusion criteria^14^ were used for CI patients: (a) all patients must meet the diagnostic requirements for CI in the Diagnostic and Statistical Manual of Mental Disorders, Fourth Edition (DSM-IV); (b) patients complained of difficulty falling asleep, difficulty maintaining sleep, or waking up early for at least 1 month; (c) patients had no other sleep disorders; (d) the patients were younger than 60 years of age; (e) psychoactive drugs were not used for patients at least 2 weeks before and during this study; (f) patients were assessed as right-handed using the Edinburgh Handedness Inventory. Exclusion criteria were as follows: (a) patients with abnormal signal in any region of the brain confirmed by conventional T1-weighted or T2 fluid-attenuated inversion recovery magnetic resonance imaging; (b) insomnia caused by organic diseases or serious mental diseases secondary to depression or general anxiety; (c) other sleep disorders; (d) pregnant, lactating or menstruating women. Forty-seven healthy controls (15 males and 32 females, age 39.85 ± 8.97 years) were recruited to meet the following criteria: (a) an Insomnia Severity Index score of less than 7; (b) no history of shift work or sleep complaints; (c) no drug or substance abuse for at least 2 weeks prior to and during the study, such as caffeine, nicotine, or alcohol; (d) no brain injury or prior severe head trauma, as confirmed by conventional T1-weighted or T2 fluid-attenuated inversion recovery MR imaging; (e) no history of psychiatric or neurological disease; (f) right-handed dominant.

Several questionnaires were completed by the study participants. These questionnaires included the insomnia severity index (ISI), Pittsburgh sleep quality index (PSQI), self-rating anxiety scale (SAS), and self-rating depression scale (SDS). The demographic and scale data of all the study participants are listed in Table 1.

**Table 1:** Demographic and scale data of all study participants.

	CI (49)	HC (47)	p value
Gender (F/M)	28/21	32/15	0.29^1^
Age (year)	39.27 ± 11.00	39.85 ± 8.97	0.61^#^
Education	9.33 ± 5.88	8.34 ± 4.43	0.18^#^
ISI	19.67 ± 3.20	7.17 ± 2.58	< 0.001^#^
PSQI	12.80 ± 3.28	5.21 ± 2.77	< 0.001^#^
SAS	51.78 ± 10.6	40.02 ± 6.13	< 0.001^#^
SDS	55.10 ± 8.58	39.89 ± 9.21	< 0.001^#^

Unless otherwise noted, data are presented as mean ± standard deviation.

^1^The P value was obtained using the chi-square test.

^#^The P value was obtained using Wilcoxon rank sum tests.

*CI* chronic insomnia, *HC* healthy control, *ISI* insomnia severity index, *PSQI* Pittsburgh sleep quality index, *SAS* self-rating anxiety scale, *SDS* self-rating depression scale.

### Data acquisition

Functional magnetic resonance imaging was performed in Medical Imaging Department of Guangdong Second Provincial General Hospital using a 1.5 Tesla MRI scanner (Achieva Nova-Dual; Philips, Best, the Netherlands)^14^. Participants were asked to rest with their eyes closed, to remain still and not fall asleep. Functional MR images were obtained in approximately 10 min using a gradient echo planar imaging (EPI) sequence as follows: interlaced scan, repetition time/echo time = 2500 ms/50 ms, section thickness = 4 mm, intersection gap = 0.8 mm, matrix = 64 × 64, field of view = 224 mm × 224 mm, flip angle = 90°, 27 axial slices, 240 volumes. After the scan, all subjects were asked if they fell asleep during the scan. Those subjects who were asleep were excluded.

### Data pre-processing

Pre-processing of the resting-state fMRI data was carried out using the Data Processing Assistant for Resting-State fMRI (DPARSF; Chao-Gan and Yu-Feng, which is based on Statistical Parametric Mapping (SPM12, http://www.fil.ion.ucl.ac.uk/spm)^18^. The first 10 image points for each participant were removed to eliminate the effects of an uneven magnetic field at the beginning or the discomfort of the test on the image quality and results. Because the MR image was scanned layer by layer, the layers had different acquisition times. Slice timing correction was used to ensure that the acquisition times for all the voxels in a volume were theoretically consistent. Subsequently, the data were corrected for any slight head movement of the participant during data acquisition. None of the participants had more than 3.0 mm of maximal displacement and 3.0 of maximal rotation in any direction. The nuisance variables included 24 head motion parameters, as well as white matter and CSF signals, and global signals were regressed out from the fMRI data. Then, spatial normalisation was conducted according to the standard Montreal Neurologic Institute template, and the data were resampled using a voxel size of 3 × 3 × 3 mm^3^. These images were smoothed by convolution using an isotropic Gaussian kernel (full width at half maximum, 4 mm). Finally, to reduce the effects of low-frequency drift and high-frequency noise, the smooth imaging data were processed to eliminate linear trends and filter over time (bandpass, 0.01–0.1 Hz).

### Data analysis

The ALFF feature^19^ can be used to analyse the amplitudes of the local characteristics of a brain’s blood oxygenation level-dependent MRI signal activity. The ALFF analysis was implemented as follows. First, the fast Fourier transform (FFT) algorithm was used to convert the time-domain signal into the frequency domain to obtain the power spectrum. The average square root of the power spectrum was the ALFF. In this study, the voxel-wise ALFF feature of each participant was calculated before filtering. In short, the time series of each given voxel was first converted to the frequency domain using the FFT. The square root of the power spectrum was calculated and averaged over a range of 0.01–0.1 Hz at each voxel. This average square root was called the ALFF of each voxel. For standardisation purposes, the ALFF of each voxel was divided by the global average ALFF value for each individual. The normalised ALFF value for each given voxel reflected the relationship between its original ALFF value and the global average ALFF value for the brain.

FC analysis examines temporal correlation in the blood oxygenation level-dependent signal changes between different regions of the brain. In this study, 116 brain regions of the AAL template were selected as seed points, and the correlation coefficients between various sub-points and other voxels of the brain were calculated to find strong time correlations with these seed points. The brain region indicates that there is a functional connection between the brain region and the brain region where the seed point is located. This method was first proposed by Biswal et al.^20^. The FC characteristics of each brain region were calculated after data pre-processing.

All of the features were calculated using the Data Processing Assistant for Resting-State fMRI (DPARSF; Chao-Gan and Yu-Feng; http://www.restfmri.net)^21^, which is based on Statistical Parametric Mapping (SPM12, http://www.fil.ion.ucl.ac.uk/spm).

All two whole-brain voxel-wise features mentioned above were converted to z-scores using Fisher’s r-to-z transformation.

### Statistical analysis and machine learning

Demographic and scale data for all study participants were analysed using SPSS (version 20; SPSS, Chicago, III). The Wilcoxon rank-sum test was used to compare the differences in age, education level, ISI, PSQI, SAS and SDS scores between CI patients and HC. Age-related differences were assessed using the chi-square test. Table 1 listed demographic and scale data for all study participants.

An LR method was developed to train a machine learning model for classification of the CI patients and HC. This classification model used a 20 rounds fivefold cross-validation method to split all the data into training samples (44 CI patients and 42 HC patients) and independent validation samples (5 CI patients and 5 HC patients). To prevent overfitting, a two-sample t-test method was used on the training samples for feature selection, and the statistically significant voxel positions were obtained, which were used to extract the corresponding features for each subject (in both the training set and validation set) for classification. The LR classifier was trained using these statistically significant features. Then, the final trained LR classifier model was used to classify for single‑shot testing on the independent validation data to acquire the classification performances (i.e. the accuracy, sensitivity, specificity, and area under the ROC curve (AUC)). All the machine learning processes for training and validation were executed in sklearn toolbox from Python.

Nonparametric permutation tests estimated the statistical significance of the average classification performances by determining whether they exceeded the level of opportunity. The class labels of the training data were randomly ranked 1000 times before training, and the 20 rounds of the fivefold CV procedure were repeated. The p value of the permutation test was defined as ${P}_{\mathrm{permutation}}=({N}_{\mathrm{exceeds}}+1)/({N}_{\mathrm{substitution}}+1)$. Here, ${N}_{\mathrm{exceeds}}$ represents the number of times the permuted performance exceeded that obtained for the true labels. ${N}_{\mathrm{substitution}}$ represents the rounds of permutation.

In the CI group, a correlation analysis (multiple comparison correction—Bonferroni correction, p < 0.05/4) was conducted to determine whether the features contributing to the prediction were correlated with clinical characteristics, i.e. the ISI, PSQI, SAS, and SDS.

## Results

### Demographic and scale data results

As listed in Table 1, there were no significant differences between the CI patients and the control group in terms of age (p = 0.61), gender (p = 0.29), and education level (p = 0.18). However, the CI patients had higher ISI, PSQI, SAS, and SDS scores (all p < 0.001) compared to the HC.

### Machine learning results

**Figure 1:**
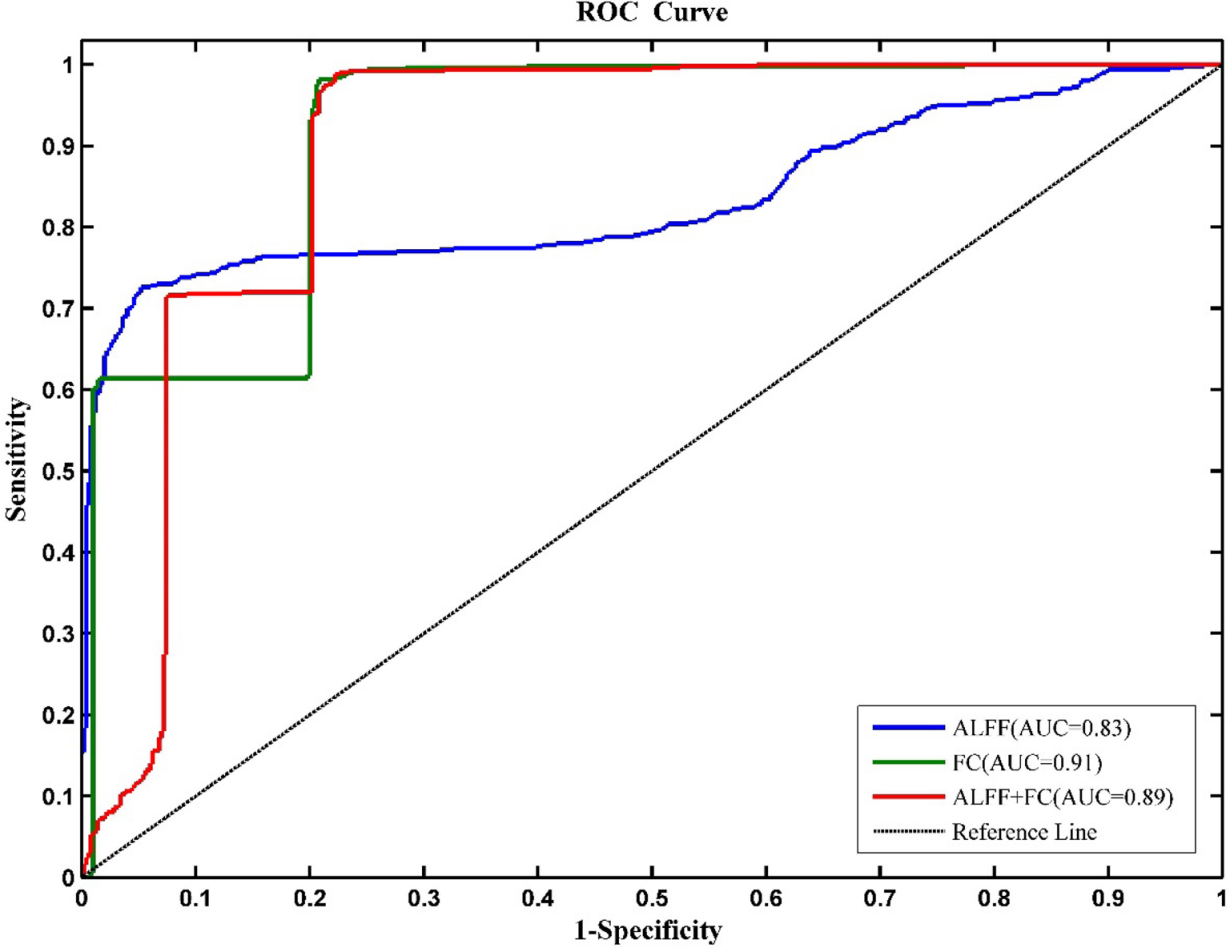
ROC curves of LR models based on different features for classification of CI patients and HC.

As shown in Table 2 and Fig. 1, LR model was developed based on the ALFF or FC features. The ALFF features provided an accuracy of 83.00%, a sensitivity of 70.00%, a specificity of 96.00%, and an AUC of 0.83. The FC features provided an accuracy of 86.60%, a sensitivity of 93.40%, a specificity of 79.80%, and an AUC of 0.91. Combining the ALFF features and FC features also showed good discrimination, with an accuracy of 86.40%, a sensitivity of 93.00%, a specificity of 79.80%, and an AUC of 0.89 (all ${P}_{\mathrm{permutation}}$< 0.001).

**Table 2:** Classification results of CI-HC.

Features	Accuracy (%)	Sensitivity (%)	Specificity (%)
ALFF	83.00	70.00	96.00
FC	86.60	93.40	79.80
ALFF + FC	86.40	93.00	79.80

**Figure 2:**
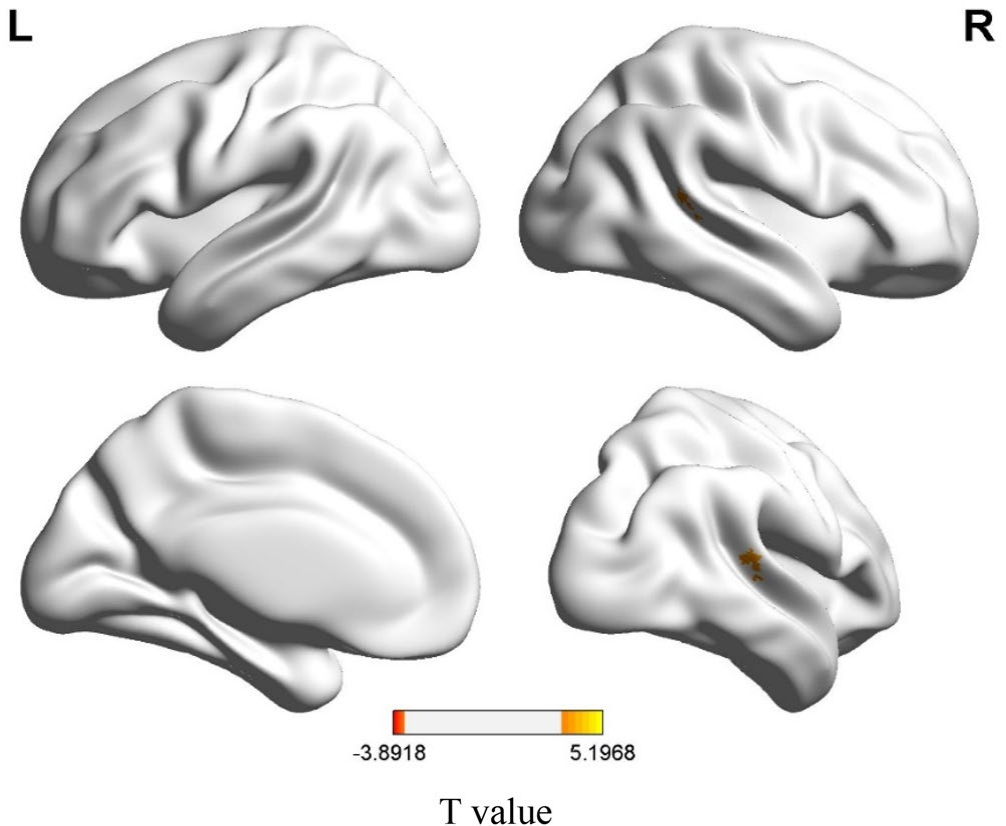
Differences in whole-brain voxel-wise ALFF features between CI patients and HC. The thresholds were p < 0.001 at the voxel level and p < 0.05 at the cluster level with GRF corrections for multiple comparisons. The colour bar represents the t value.

**Figure 3:**
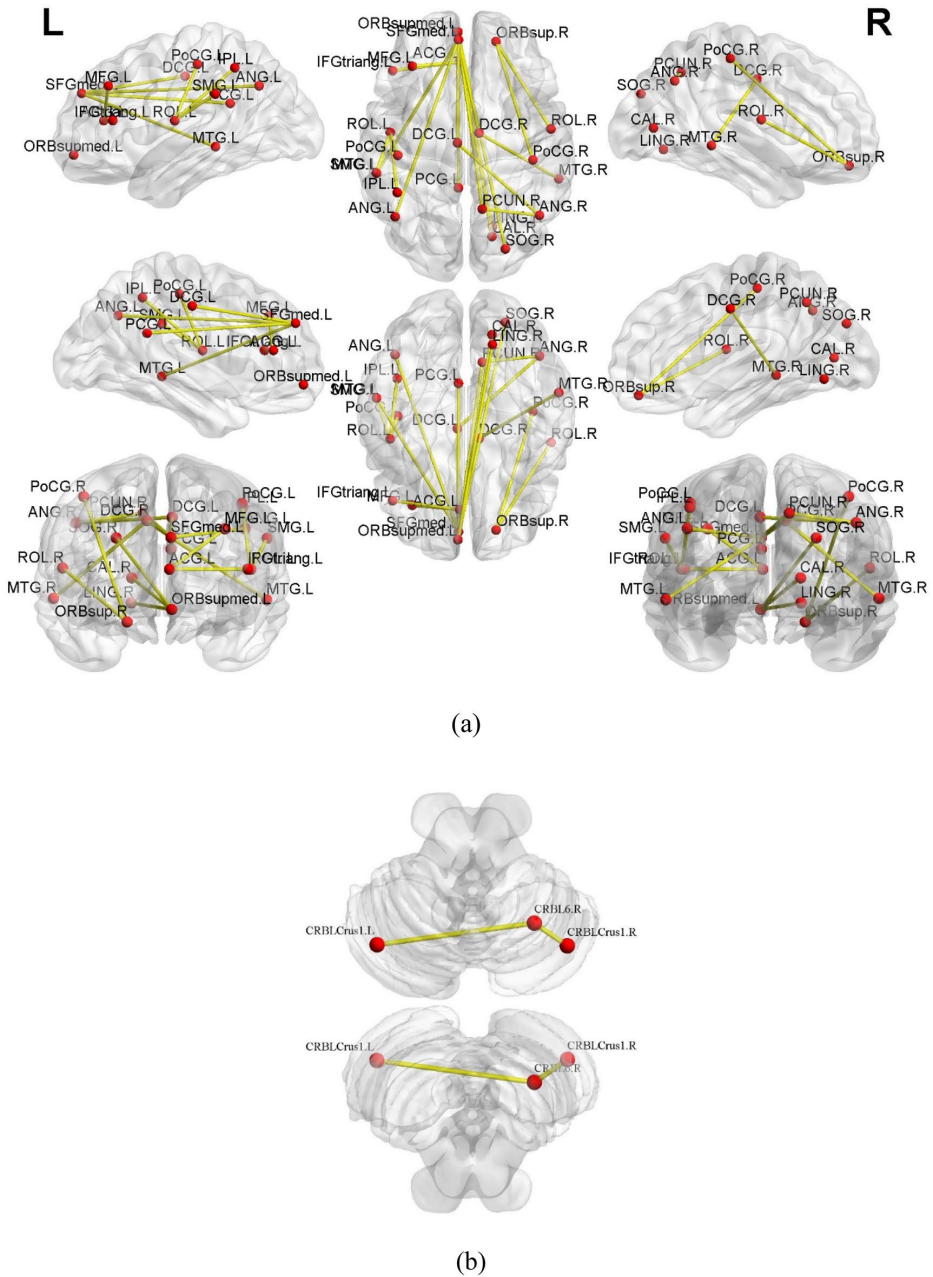
Visualisation of Table 3 created from brain region point of view: (**a**) decreased functional connectivities in CI patients and (**b**) increased functional connectivities in CI patients.

Figures 2 and 3 display the regions showing between-group differences in the whole-brain voxel-wise ALFF features and whole-brain voxel-wise FC features, respectively (multiple comparison correction—GRF, p-voxel < 0.001, p-cluster < 0.05). The estimated Gaussian filter widths (FWHM, in millimetres) were [7.371, 7.291, 6.984]. As shown in Fig. 2, CI patients had higher ALFF values mainly in the superior temporal gyrus and middle temporal gyrus. Compared with the HC, patients with insomnia showed decreased functional connectivities among widespread regions, including the orbital part of the superior frontal gyrus, middle frontal gyrus, triangular part of the inferior frontal gyrus, rolandic operculum, medial superior frontal gyrus, orbital part of the middle frontal gyrus, anterior cingulate, paracingulate gyrus, median cingulate, paracingulate gyrus, posterior cingulate gyrus, calcarine fissure and surrounding cortex, lingual gyrus, superior occipital lobe, postcentral gyrus, inferior parietal gyrus, supramarginal gyrus, angular gyrus, praecuneus, middle temporal gyrus, superior cerebellum, and part of the cerebellum. These regions belong to the some functional connectivity networks, including those between the orbital part of the frontal lobe (ORB) and Rolandic operculum (ROL), postcentral gyrus (PoCG), sensory-motor network (SMN), lingual gyrus (LING), and calcarine fissure and surrounding cortex (CAL); between the ROL and PoCG, SMN, and CEN; between the DMN and DMN, CEM, median cingulated, and paracingulate gyrus (DCG); between the SN and DMN; between the AN and DMN; and between the DCG and the CEM and AN. In addition, increased functional connectivity was found between the cerebellum (CER) and the cerebellum (CER), occipital lobe, and lingual gyrus in CI patients. Details can be seen in Table 3 and Fig. 3a,b.

**Table 3:** Decreased and increased functional connectivities in CI patients (multiple comparison correction—GRF, p-voxel < 0.001, p-cluster < 0.05).

Connected regions	Network	z value in CI patients	z value in HC	Two-sample 2-tailed t-tests	p value
Frontal_Sup_Orb_R-Rolandic_Oper_R	ORB-ROL	0.06 ± 0.10	0.16 ± 0.14	− 3.79	0.00027
Frontal_Sup_Orb_R-Postcentral_R	ORB-PoCG	0.69 ± 0.13	0.18 ± 0.16	− 3.65	0.00043
Rolandic_Oper_L-Postcentral_L	ROL-PoCG	0.24 ± 0.16	0.40 ± 0.16	− 3.92	0.00017
Rolandic_Oper_L-SupraMarginal_L	ROL-SMN	0.29 ± 0.19	0.44 ± 0.22	− 3.67	0.00040
Rolandic_Oper_L-Parietal_Inf_L	ROL-CEN	0.19 ± 0.16	0.33 ± 0.16	− 3.89	0.00019
Frontal_Mid_Orb_L-Occipital_Sup_R	ORB-SMN	0.12 ± 0.14	0.22 ± 0.15	− 3.81	0.00025
Frontal_Mid_Orb_L-Lingual_R	ORB -LING	0.07 ± 0.13	0.15 ± 0.14	− 3.85	0.00022
Frontal_Mid_Orb_L-Calcarine_R	ORB -CAL	0.11 ± 0.12	0.21 ± 0.12	− 3.82	0.00024
Frontal_Sup_Medial_L-Precuneus_R	DMN-DMN	0.24 ± 0.14	0.35 ± 0.15	− 3.79	0.00027
Frontal_Sup_Medial_L-Precuneus_L	DMN-DMN	0.35 ± 0.17	0.49 ± 0.14	− 3.81	0.00025
Frontal_Sup_Medial_L-Cingulum_Mid_R	DMN-DCG	0.30 ± 0.15	0.42 ± 0.13	− 3.86	0.00021
Frontal_Sup_Medial_L-Cingulum_Mid_L	DMN-DCG	0.36 ± 0.17	0.50 ± 0.15	− 4.18	0.00007
Cingulum_Ant_L-Frontal_Mid_L	SN-DMN	0.08 ± 0.11	0.20 ± 0.16	− 3.80	0.00026
Cingulum_Ant_L-Frontal_Inf_Tri_L	SN-IFGtriang	0.05 ± 0.11	0.18 ± 0.15	− 3.89	0.00019
Cingulum_Ant_L-Frontal_Inf_Oper_L	SN-IFGoperc	0.02 ± 0.15	0.16 ± 0.19	− 3.78	0.00028
Cingulum_Mid_L-Parietal_Inf_R	DCG-CEM	0.21 ± 0.12	0.32 ± 0.15	− 3.82	0.00024
Cingulum_Mid_R-Temporal_Mid_R	DCG-AN	0.15 ± 0.14	0.31 ± 0.16	− 3.95	0.00015
Cingulum_Post_L-Frontal_Sup_Medial_L	PCG-DMN	0.27 ± 0.13	0.42 ± 0.16	− 4.00	0.00013
Precuneus_R-Angular_R	DMN-DMN	0.24 ± 0.08	0.36 ± 0.13	− 3.69	0.00038
Precuneus_R-Parietal_Inf_R	DMN-CEN	0.24 ± 0.13	0.37 ± 0.16	− 3.65	0.00043
Angular_L-Frontal_Sup_Medial_L	DMN-DMN	0.18 ± 0.15	0.34 ± 0.14	− 4.02	0.00012
Angular_L-Frontal_Sup _L	DMN-DMN	0.18 ± 0.17	0.34 ± 0.18	− 3.80	0.00026
Temporal_Mid_L-Frontal_Sup_Medial_L	AN-DMN	0.11 ± 0.09	0.21 ± 0.11	− 3.99	0.00013
Cerebelum_6_R-Cerebelum_Crus1_L	CER-CER	0.47 ± 0.26	0.27 ± 0.27	3.52	0.00067
Cerebelum_6_R-Cerebelum_Crus1_R	CER-CER	0.51 ± 0.23	0.30 ± 0.22	3.92	0.00017

*CI* chronic insomnia, *HC* healthy control, *L* left, *R* right, *DMN* default mode network, *SN* salience network, *AN* affective network, *CEN* central-executive network, *SMN* sensory-motor network, *CER* cerebellum, *Frontal_Sup_Orb (ORB)* superior frontal gyrus orbital part, *Frontal_Mid* middle frontal gyrus, *Frontal_Inf_Tri (IFGtriang)* inferior frontal gyrus, triangular part, *Frontal_Inf_Oper_L (IFGoperc)* inferior frontal gyrus, opercular part, *Rolandic_Oper (ROL)* rolandic operculum, *Frontal_Sup_Medial* medial superior frontal gyrus, *Frontal_Mid_Orb (ORB)* middle frontal gyrus orbital part, *Cingulum_Ant* anterior cingulate paracingulate gyrus, *Cingulum_Mid (DCG)* median cingulate paracingulate gyrus, *Cingulum_Post* posterior (PCG), cingulate gyrus, *Calcarine (CAL)* calcarine fissure and surrounding cortex, *Lingual (LING)* lingual gyrus, *Occipital_Sup* superior occipital lobe, *Postcentral (PoCG)* postcentral gyrus, *Parietal_Inf* Inferior parietal gyrus, *Supra Marginal* supramarginal gyrus, *Angular* angular gyrus, *Precuneu*s precuneus, *Temporal_Mid* middle temporal gyrus, *Cerebelum_6* Cerebellum_Superior, *Cerebelum_Crus1* part of cerebellum.

### Correlation analysis results

In the CI group, the correlation analysis results showed that the FC between the DMN and DMN, and between the DMN and DCG, were positively correlated with the ISI. Otherwise, the SDS was negatively related to the FC between the DCG and CEM. All correlation analysis results can be seen in Table 4.

**Table 4:** Associations of FC features and clinical characteristics in CI patients.

Connected regions in CI patients	Network	ISI		SDS	
		r	p value	r	p value
Frontal_Sup_Medial_L–Precuneus_R	DMN-DMN	0.48	0.00047		
Frontal_Sup_Medial_L–Cingulum_Mid_R	DMN-DCG	0.38	0.00749		
Cingulum_Mid_L–Parietal_Inf_R	DCG-CEM			− 0.39	0.00620

*CI* chronic insomnia, *ISI* insomnia severity index, *SDS* self-rating depression scale; for other abbreviations please see Table 2.

## Discussion

This study investigated whether the ALFF and FC features could be used as neurological markers for the classification of CI. LR was used for the classification of the CI-HC group. The results showed that combined ALFF and FC features had good discrimination, with an accuracy of 86.40%, a sensitivity of 93.00%, a specificity of 79.80%, and an AUC of 0.89. In addition, seen in Table 4, the correlation analysis results suggested that some of the FC among related regions was positively correlated with the ISI and negatively correlated with the SDS.

Several previous findings^9,11,14,18,21–23^ based on fMRI suggested that spontaneous neural activity in the anterior cingulate, prefrontal cortex, and orbital part of the frontal lobe was disrupted in patients with insomnia. In line with these findings, this research found that when using these regions as seed regions of interest, the seed-based, voxel-wise FC metrics also differed between the CI patients and HC. In addition, previous findings^9,24,25^ generally suggested that the metabolism or spontaneous neural activity in the prefrontal cortex was reduced. Therefore, it was reasonable to explain why CI patients had decreased FC between the left superior frontal gyrus and the other brain regions with the left superior frontal gyrus as the seed region.

Specifically, the classification performances of FC features were excellent for diagnosing CI patients in this study (accuracy: 86.60%, sensitivity: 93.40%, specificity: 79.80%, and AUC: 0.91). Statistically significant FC features were found in many brain regions such as the anterior cingulate, prefrontal cortex, orbital part of the frontal lobe, angular gyrus, cingulate gyrus, praecuneus, parietal lobe, and temporal gyrus. From the perspective of a functional connection network^9–12^, these regions involve the DMN, SN, SMN, AN, and CEN. Consistent with previous studies, this study suggested that regions associated with wakefulness, mood, anxiety/contemplation, significant/attention, and sensorimotor activity showed significantly decreased interactions with each other in CI patients. However, in this study, for the seed voxels selected in Cerebelum_6_R (AAL template ROI 100) (Cerebellum_Superior), the CI patients had significantly increased FC values in Cerebelum_Crus1_L, Cerebelum_Crus1_R, occipital lobe, and lingual gyrus. One explanation might be that the increased FC with the cerebellum as the seed region was compensatory to the dysfunction in the cerebellum.

In a study by Li et al.^5^, CI patients displayed lower ALFF values in the bilateral cerebellum posterior lobes, with higher ALFF values in the right middle/inferior temporal lobe extended to the right occipital lobe. Compared with normal controls, Dai et al.^7^ also found that CI patients had higher ALFF values in the temporal and occipital lobes, with lower ALFF values in the bilateral cerebellum. This was consistent with the findings in this study that the ALFF features had statistical differences mainly in the superior temporal gyrus and middle temporal gyrus between CI patients and HC. The statistical ALFF features were also good for CI-HC classification with an accuracy of 83.00%, a sensitivity of 70.00%, a specificity of 96.00%, and an AUC of 0.83.

To the best of our knowledge, few studies have applied machine learning methods to the automatic classification of CI patients using resting-state metrics (FC, ALFF). Li et al.^18^ suggested that the FC strength (FCS) could be potential neuromarkers for the classification of CI patients and HC using the support vector machine (SVM) method. The classification performance included an accuracy of 81.5%, a sensitivity of 84.9%, a specificity of 79.1%, and an AUC of 83.0%. In this study, an LR model was developed for the classification of CI patients and HC and also showed better discrimination which proved that these two features can be used as neurological markers for the diagnosis of insomnia.

This study had several limitations. First, it was assumed that the participants represented a homogeneous sample of individuals with a single insomnia condition. However, it is increasingly believed that insomnia may be a heterogeneous disease. Therefore, if different studiesinclude different proportions of each subtype^24,25^, which may not be identified, this may lead to inconsistent findings. Second, the model parameter tuning used in this study used the method of grid optimisation. The grid optimisation method adopts an exhaustive method and traverses all possible combinations of parameters. Thus, it is not fast. Faster parameter optimisation methods such as genetic algorithms^26^ will be adopted in the future to improve the efficiency of the algorithm. Third, this study only used logistic regression as a machine learning method. Combining different machine learning methods would help to improve the model performance. Fourth, only functional MR imaging data were used. The integration of structural and functional data may be a more effective method to elucidate disease factors that are shared across different metrics. Fifth, the participants in the present study were all right-hand dominant; therefore, it was not possible to identify the relationship between the R-sided and L-sided findings with handedness. Sixth, only the static characteristics of the traditional (low-order) FC were studied, not their dynamic characteristics. The ‘correlation of correlation’^27,28^ generates high-order functional connectivity (HOFC) based on the FC dynamics, which characterises higher-level brain functional interactions and supplements traditional (low-order) FC. HOFC has been successfully applied to early mild cognitive impairment (MCI) detection and has shown superior performance compared with the low-order FC-based methods^29,30^. Further research using HOFC is required when using machine learning methods for CI.

## Conclusion

In summary, despite these limitations, the results of this study showed that ALFF features and FC features had excellent performance for diagnostic identification of chronic insomnia using logistic regression approach and might serve as potential neuromarkers for CI. This proposed methodology could be applied in clinical practice for diagnostic identification of CI.

## Data Availability

Datasets generated and/or analysed during the current study are not publicly available due the relevant regulations of our hospital (Guangdong Second Provincial General Hospital) but are available from the corresponding author on reasonable request.
